# Are all fibrinogen concentrates the same? The effects of two fibrinogen therapies in an afibrinogenemic patient and in a fibrinogen deficient plasma model. A clinical and laboratory case report

**DOI:** 10.3389/fmed.2024.1391422

**Published:** 2024-05-30

**Authors:** Soutiam Goodarzi, Jeries Abu-Hanna, Sarah Harper, Dalia Khan, Gael Morrow, Nicola Curry

**Affiliations:** ^1^Oxford University Medical School, Medical Sciences Division, John Radcliffe Hospital, Oxford, United Kingdom; ^2^Radcliffe Department of Medicine, Oxford University, Oxford, United Kingdom; ^3^Oxford Haemophilia and Thrombosis Centre, Oxford University Hospitals National Health Service (NHS) Foundation Trust, Oxford, United Kingdom; ^4^School of Pharmacy and Life Sciences, Robert Gordon University, Aberdeen, United Kingdom

**Keywords:** afibrinogenemia, fibrinogen concentrate, hemostasis, inherited bleeding disorder, fibrinolysis

## Abstract

The choice of treatments for inherited, or acquired, fibrinogen deficient states is expanding and there are now several fibrinogen concentrate therapies commercially available. Patients with the rare inherited bleeding disorder, afibrinogenemia, commonly require life-long replacement therapy with fibrinogen concentrate to prevent hemorrhagic complications. Recent reports in the setting of acquired bleeding, namely trauma hemorrhage, have highlighted the potential importance of the different compositions of fibrinogen supplements, including cryoprecipitate and the various plasma- derived concentrates. Clot strength and the subsequent susceptibility of a clot to lysis is highly dependent on the amount of fibrinogen as well as its structural composition, the concentration of pro- and anti-coagulant factors, as well as fibrinolytic regulators, such as factor XIII (FXIII). This report details the effects of two commercially available fibrinogen concentrates (Riastap^®^, CSL Behring and Fibryga^®^, Octapharma) on important functional measures of clot formation and lysis in a patient with afibrinogenemia. Our report offers insights into the differential effects of these concentrates, at the clot level, according to the variable constituents of each product, thereby emphasizing that the choice of fibrinogen concentrate can influence the stability of a clot *in vivo*. Whether this alters clinical efficacy is yet to be understood.

## Introduction

Fibrinogen (Fg) is the main architectural component of a blood clot (1). Following its cleavage by thrombin, soluble Fg is converted into insoluble fibrin monomers which can self-polymerize. The resultant protofibrils form a fibrin mesh at sites of injury (2). Clot formation, clot structure and clot stability are influenced by the interplay of many factors during the process of fibrin formation. These influences can broadly be thought of as: (1) the effects of pro-/anti-coagulant and fibrinolytic regulatory proteins directly affecting either thrombin generation or fibrinolysis; (2) the natural variety in Fg structure (e.g., relative amounts of gamma-prime; post-translational modifications); (3) the effects of blood flow and the cell surface (e.g., endothelial, platelet) (3). The complex interplay of these interactions results in variations in the diameter, and density, of the fibrin strands that form, which affects clot strength and subsequently the susceptibility of the clot to lysis (2).

Fibrinogen deficiency is most often encountered clinically in an acquired setting, for example, during major bleeding after traumatic injury or childbirth (4). Much less commonly, it may be the result of an inherited rare bleeding disorder, namely hypo- or afibrinogenemia, which affect 1–2 individuals per million (5). These are hereditary conditions characterized by an abnormally low production of Fg, with Clauss Fg levels 1.0 g/L or less for hypofibrinogenemia, and undetectable levels for afibrinogenemia (5). Afibrinogenemia carries a lifelong high risk of bleeding and is often managed with regular infusions of fibrinogen concentrate on a prophylactic basis.

There are several Fg concentrates available commercially. Data from the trauma setting have shown that these concentrates are compositionally different and *in vitro* and *ex vivo* work has reported variation in both clot lysis and clot structure, dependent on the Fg supplementation used (6–8). One important difference noted between the Fg concentrates has been the concentration of factor XIII (FXIII), which is much higher in Fibryga^®^ (Octapharma, Switzerland) concentrate when compared to RiaSTAP^®^ (CSL Behring, Germany). FXIII plays a vital role in cross-linking fibrin gamma chains, thus stabilizing the fibrin mesh and reducing lytic susceptibility. This may alter a patient's hemostatic response to Fg therapy.

Here we present data from a young patient with congenital afibrinogenemia who was switched from RiaSTAP^®^ to Fibryga^®^ concentrate due to an operational change in hospital prescribing. The aim of this study was to explore, in detail, the differential effects of two Fg concentrates on clot formation, stability and lysis. Further experiments, to investigate the hemostatic differences in more detail, were conducted using fibrinogen-deficient plasma spiked with each concentrate, *in vitro*.

### Case report

**Figure 1:**
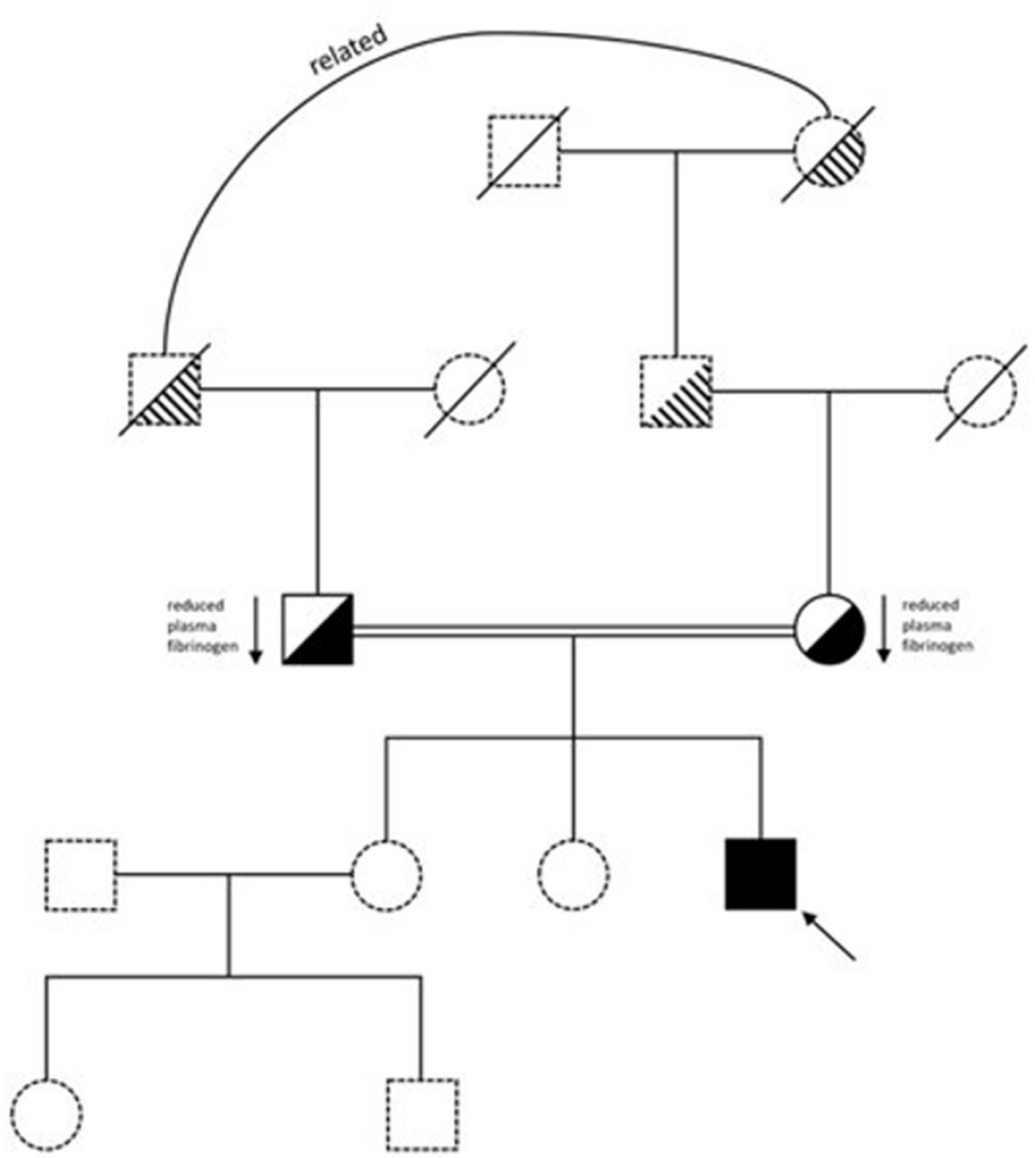
Family pedigree.

Our patient is a 26-year-old male of Pakistani descent, born to distantly related parents, who was diagnosed with congenital afibrinogenemia a few weeks after birth. He has given informed consent for his details to be presented. He was originally admitted to the Special Care Baby Unit at 4-days-old with jaundice and vomiting. There was no history of trauma or of a bleeding disorder in the family (Figure 1). He was born at 39 weeks via elective C-section. There were no known complications during pregnancy. The patient received his first dose of vitamin K immediately after birth.

He developed tonic-clonic seizures 6 h into his admission, treated with phenobarbitone, phenytoin and paraldehyde. He was diagnosed with a small germinal matrix hemorrhage on cranial ultrasound scan with no ventricular dilatation. He stabilized within a few days and was sent home. Three weeks later, at a routine check, another cranial ultrasound scan was taken due to an abnormal increase in his head circumference, upon which ventricular dilatation and parieto-occipital ischemia were noted, caused by the previous hemorrhage. Review of his blood tests showed abnormal coagulation: PT 31 s (normal range, NR: 9–12 s); APTT 41 s (NR: 20–30 s). Clauss Fg was undetectable at < 0.3 g/L, as was Fg antigen.

Congenital afibrinogenemia was suspected and considering his clinical presentation of prior intracranial bleeding, cryoprecipitate was given immediately. His trough Fg level was then maintained above 0.8 g/L, necessitating cryoprecipitate infusion every 3 days. Genetic testing confirmed the homozygous variant c.78+5G>A (Supplementary Figure S1) (9). His parents were confirmed as heterozygote for the variant, both with a low Clauss Fg level (mother-−1.3 g/L; father-−1.2 g/L), and neither had a bleeding history.

Following a good recovery at 3 months, the cryoprecipitate was discontinued. Unfortunately, he developed a second intracranial hemorrhage at 8 months of age, and since then, has received Fg concentrate prophylaxis, maintaining a trough ≥1 g/L. He has had no further bleeding episodes and was taught to self-infuse at 18-years. He has not been exposed to any significant hemostatic challenges, such as surgery, since commencing prophylaxis. In 2023, the hospital routinely switched Fg concentrate therapy due to operational changes in hospital prescribing and we undertook extended laboratory testing to compare the two licensed products. At the time of testing, the patient weighed 82.7 kg, and had a body mass index of 25.6 kg/m^2^. We report the results below.

## Methods

Venous blood samples were taken from the patient at various time points, drawn into 3.2% citrate (Becton Dickinson^®^, New Jersey, USA), after written informed consent. Trough (taken at 72 h) and peak (10 min post-dose) samples were taken for RiaSTAP^®^. A second trough sample was taken for RiaSTAP^®^ immediately prior to administration of the first Fibryga^®^ dose to delineate how much RiaSTAP^®^ had remained from the previous dose. A combined RiaSTAP^®^ + Fibryga^®^ sample was taken immediately after the first Fibryga^®^ injection. Trough (taken at 72 h) and peak (10 min post-dose) samples were taken for Fibryga^®^ several months after the switch to ensure complete clearance of any RiaSTAP^®^.

At each sample draw, the following tests were performed: Clauss Fg, Fg antigen; FXIII, alpha-2 anti-plasmin (A2AP), ROTEM EXTEM and FIBTEM (10); fibrin polymerization; plasmin generation; confocal microscopy. In some instances, these tests were conducted on increasing concentrations of RiaSTAP^®^ or Fibryga^®^ spiked into fibrinogen-deficient plasma (F1DP) (Affinity Biologicals, Canada). Briefly, Fg concentrate was reconstituted to the standard 20 g/L concentration, and in each assay, increasing volumes of Fg concentrate was added replacing the equivalent volume of buffer (assay dependent).

### Standard and extended clotting factor tests

Clauss Fg (Dade Thrombin Reagent, Siemens, Germany) (NR: 1.5–4.5 g/L), Fg antigen (LIAPHEN, Hyphen BioMed, France) (NR: 1.94–4.17 g/L), FXIII (FXIIIA, Siemens) and alpha2 antiplasmin (α2AP, Siemens) were measured with chromogenic assays: NR: 50–150% and 80–130%, respectively. All assays were analyzed using a Sysmex CS-5100 analyzer in the specialist hemostasis laboratory.

### Clot lysis

Patient or F1DP (30%), 16 μM phospholipids (Rossix, Molndal, Sweden), 45 pM tPA (NIBSC, Potters Bar, UK) in 10 mM TRIS pH 7.4 0.01% Tween20 was added to 96 well flat-bottom assay plates. In F1DP experiments an increasing dose of Fg (range: 0.5–6 g/L) was added, using RiaSTAP^®^ or Fibryga^®^. Clotting was initiated with 0.01 U/mL thrombin (Sigma Aldrich, USA), 10.6 mM CaCl_2_. Absorbance at 405 nm was recorded every 60 s for 4 h using Ascent software (version 2.6). Data were analyzed by calculating time to 50% lysis using Shiny App software.^1^

### Fibrin polymerization

Several concentrations—0.5, 1.0, 2.0, 4.0, and 6.0 g/L of RiaSTAP^®^ and Fibryga^®^ were chosen to compare polymerization rates, under standard conditions. Briefly, polymerization was activated using 0.1 U/ml thrombin and 5 mM calcium chloride, in 10 mM TRIS buffer. Turbidity was measured every 10 s for 265 min.

### Plasmin generation

10% plasma was mixed with 0.5 mM S-2251 chromogenic substrate (Chromogenix, Ohio, USA), and added to 10 nM tPA (Actilyse, Boehringer Ingelheim, Germany). Absorbance readings at 405 nm were taken every 30 s for 8 h at 37 °C. The rate of plasmin generation was determined using the Shiny App for zymogen activation.^2^

### Confocal microscopy

Clots were formed using 30% plasma (patient samples or F1DP with 3 or 6 g/L RiaSTAP^®^ or Fibryga^®^), 0.25 μM Alexa Fluor 488 (AF488) fibrinogen (ThermoFisher Scientific, USA) and 16 μM phospholipids. Clotting was initiated with 0.1 U/ml thrombin and 10.6 mM CaCl2 before adding to Ibidi μ-slide VI0.4 chambers (Ibidi GmbH, Germany). Representative images are shown in the manuscript. Images were recorded on Zeiss LSM 880. Images were analyzed using FIJI v2.15.0 and the Diameter J plug-in.

### SDS-PAGE

Proteins were boiled in NuPAGE LDS Sample Buffer (Invitrogen) and NuPAGE Sample Reducing Agent (Invitrogen) at 70°C for 10 min. Equal amounts of protein along with SeeBlue Plus 2 Pre-stained Protein Standards were loaded into wells of NuPAGE 4–12% Bis-Tris Protein Gels (Invitrogen) and run in NuPAGE MOPS SDS Running Buffer (Invitrogen) at 200 V for 50 min. Gels were stained with 0.1% (w/v) Coomassie Brilliant Blue in 10% (v/v) acetic acid, 50% (v/v) methanol, and 40% (v/v) distilled water for 3 h with shaking. Gels were washed with 10% acetic acid, 50% methanol, 40% distilled water 3 times for 2 h.

### Statistical analysis

Data are presented descriptively using mean and standard deviation. Categorical data are presented as frequencies and percentages. Clinical and laboratory measures were compared using student's *t*-test (two-group comparisons) and one-way ANOVA (three-group comparisons). A *P*-value of < 0.05 was chosen to represent statistical significance throughout. Data analysis was performed using GraphPad Prism 10, GraphPad Software LLC.

## Results

### Patient samples

The patient had no change in their clinical picture throughout this study and they did not experience any abnormal bleeding whilst receiving treatment with either Fg concentrate.

Prophylactic treatment with either concentrate, at a dose of 3 g, led to a similar incremental recovery: Clauss Fg rose from 0.9 to 2.0 g/L, Riastap^®^ and 0.9 to 2.1 g/L, Fibryga^®^. Trough Clauss Fg was higher following Fibryga^®^ infusion (1.4 g/L), compared to RiaSTAP^®^ (0.9 g/L). FXIII levels were higher following Fibryga^®^ infusion (1.59 IU/mL), vs. 0.96 IU/mL, RiaSTAP^®^, reflecting previously reported higher FXIII levels in Fibryga^®^ (11, 12). EXTEM and FIBTEM ROTEM showed an expected increment in the maximum amplitudes, with no differences between the two products. FIBTEM maximum amplitude (MA) rose from 8 to 14 mm (after 3 g RiaSTAP^®^) and from 10 to 15 mm (after 3 g Fibryga^®^). At the same timepoints, EXTEM MA rose 57 to 63 mm (Riastap^®^) and 59 to 66 mm (Fibryga^®^). 6% lysis was detected in the Pre-Fibryga^®^ FIBTEM sample, otherwise all samples showed 0 or 1% lysis.

At the trough sample timepoints, lysis was quicker in the Fibryga^®^ sample (79.6 min) when compared to Riastap^®^ (90.3 min) *p* < 0.0001 (Supplementary Figure S2A). 50% clot lysis times prolonged after both treatments. However, the increase with Fibryga^®^ (~50% longer, to 119 min) was significantly greater than after Riastap^®^ (~7% longer, to 97 min) (Supplementary Figure S2B). Clot turbidity rose after both treatments, as expected, but the change was less with RiaSTAP^®^ 0.095 (trough) to 0.098 (peak) compared with 0.0133 Fibryga^®^ (trough) to 0.125 (peak). The first trough Riastap^®^ sample was hemolyzed, which may have affected these results.

**Figure 2:**
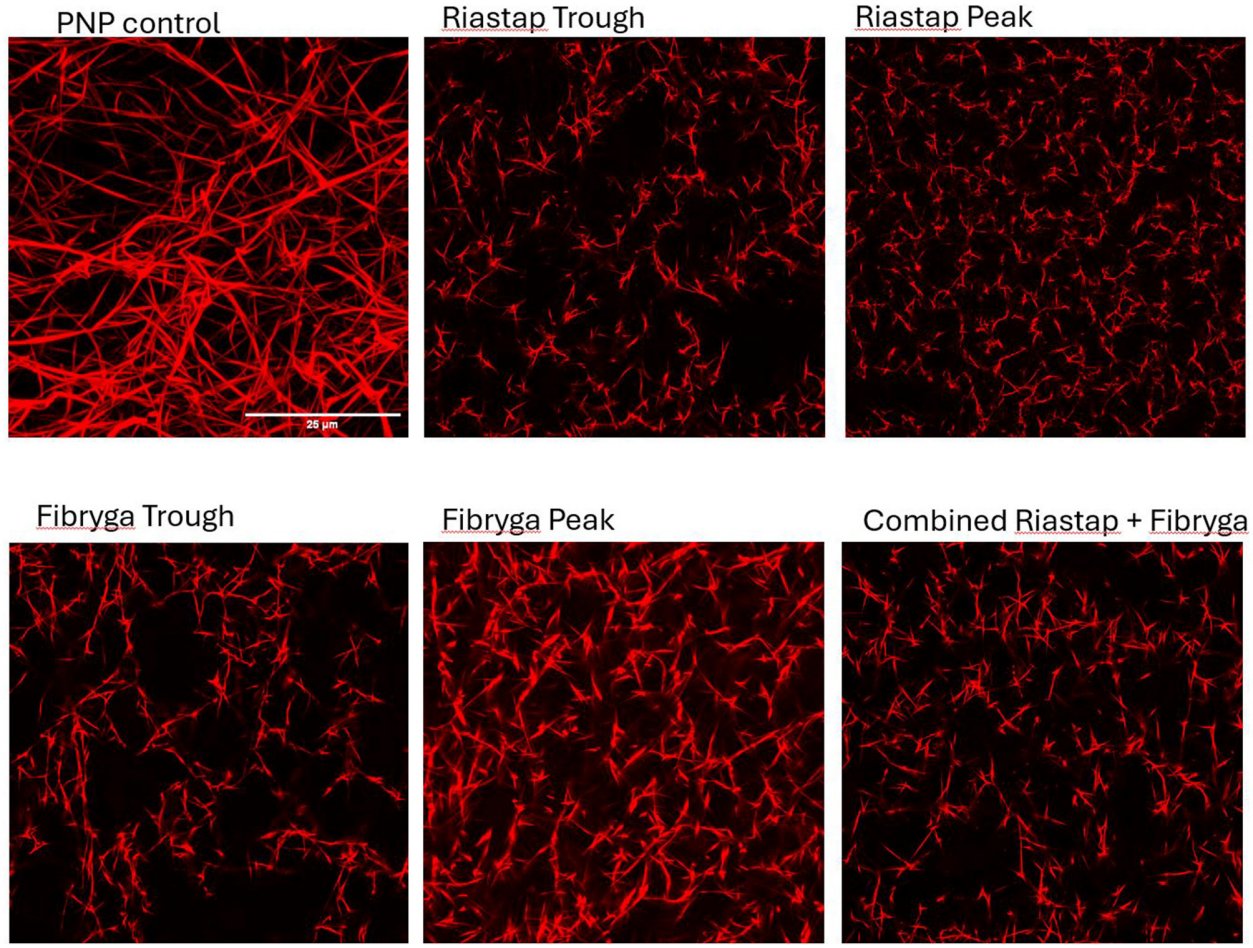
Confocal imaging of patient samples. Confocal microscopy, Alexa Fluor 488 (AF488) fibrinogen, delineating fibrin strands in: pooled normal plasma (PNP) as a control; Riastap^®^ trough and peak samples; Fibryga^®^ trough and peak samples and the “combined sample”—a mix of Riastap^®^-Fibryga^®^.

To evaluate these fibrinolytic differences further and explore fibrin clot structure, we performed confocal microscopy (Figure 2). Compared to PNP, the fibrin fibers in most patient samples were shorter and thinner. Treatment with Riastap^®^, or when the Fibryga^®^ dose was given for the first time (e.g., with Riastap^®^ still present), led to no change in diameter or length of fibrin fibers. Fibryga^®^ treatment led to an increase in fiber length, with fibers showing a greater diameter, when compared to pre-treatment. There was a reduction in numbers of pores after treatment with both concentrates.

### *In vitro* results, reconstituted fibrinogen concentrates

Factor levels for RiaSTAP^®^ and Fibryga^®^ concentrates are shown in Supplementary Table S1. Clauss Fg and antigen levels were comparable. A2AP levels were low in both products, Fibryga^®^ (4%), RiaSTAP^®^ (1%). Plasminogen levels were comparably low. FXIII levels were higher in Fibryga^®^ (1.24 IU/mL) vs. negligible, RiaSTAP^®^ (0.01 IU/mL), as previously reported (6).

Fibrin polymerization rates with RiaSTAP^®^ were slower, across all Fg concentrations (Supplementary Figure S3). Fibrin polymerization was fastest at 2 g/L for both concentrates (772.51 Abs/s RiaSTAP^®^ and 1,879.88 Abs/s Fibryga^®^). Maximum turbidity was lower with RiaSTAP^®^ across all concentrations (0.12 RiaSTAP^®^ vs. 0.46 Fibryga^®^ at 0.5 g/L; 1.52 RiaSTAP^®^ vs. 1.71 Fibryga^®^ at 6 g/L), though the difference between the two products was much less at the higher concentrations.

**Figure 3:**
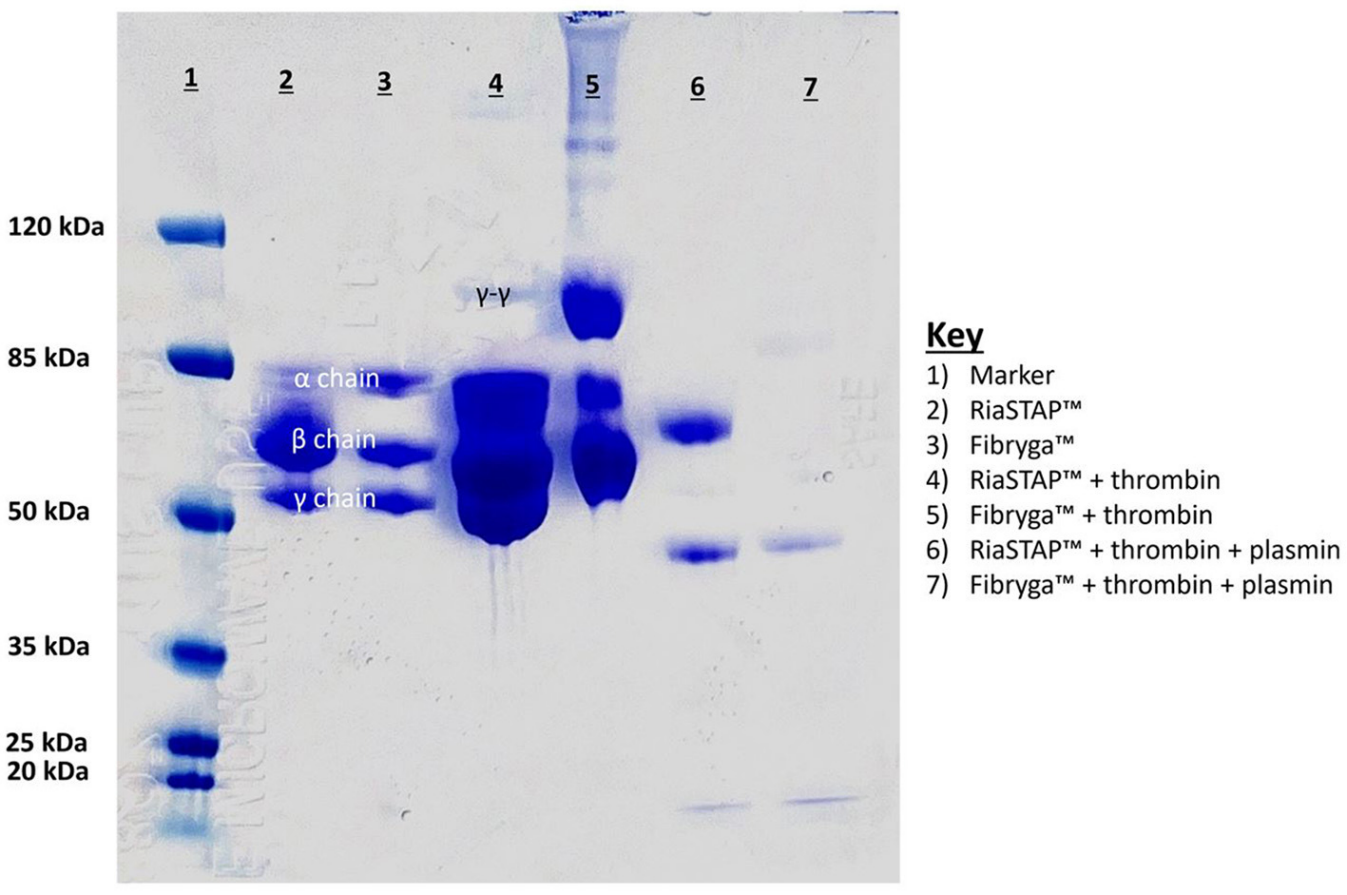
SDS Page analysis of reconstituted fibrinogen concentrates. Lanes of gel: 1. Ladder; 2. Riastap^®^; 3. Fibryga^®^; 4. Thrombin + Riastap^®^; 5. Thrombin + Fibryga^®^; 6. Thrombin + plasmin + Riastap^®^; 7. Thrombin + plasmin + Fibryga^®^.

SDS-Page analysis (Figure 3) of the reconstituted concentrates revealed differences. RiaSTAP^®^ had uneven distribution of chains with a strong signal for the β chain and a lower signal for the α chain, unlike the Fibryga^®^ product which produced an equal signal for the α, β, and γ chains. Addition of thrombin resulted in relatively small amounts of γ-γ crosslinks for RiaSTAP^®^. The γ chain signal was reduced for Fibryga^®^ on exposure to thrombin, with marked increases in γ-γ cross-linking, likely because of the higher FXIII levels. There was incomplete fibrin breakdown with Riastap^®^ as the signal at the β position remained after plasmin treatment.

### *In vitro* results, fibrinogen-deficient plasma

**Figure 4:**
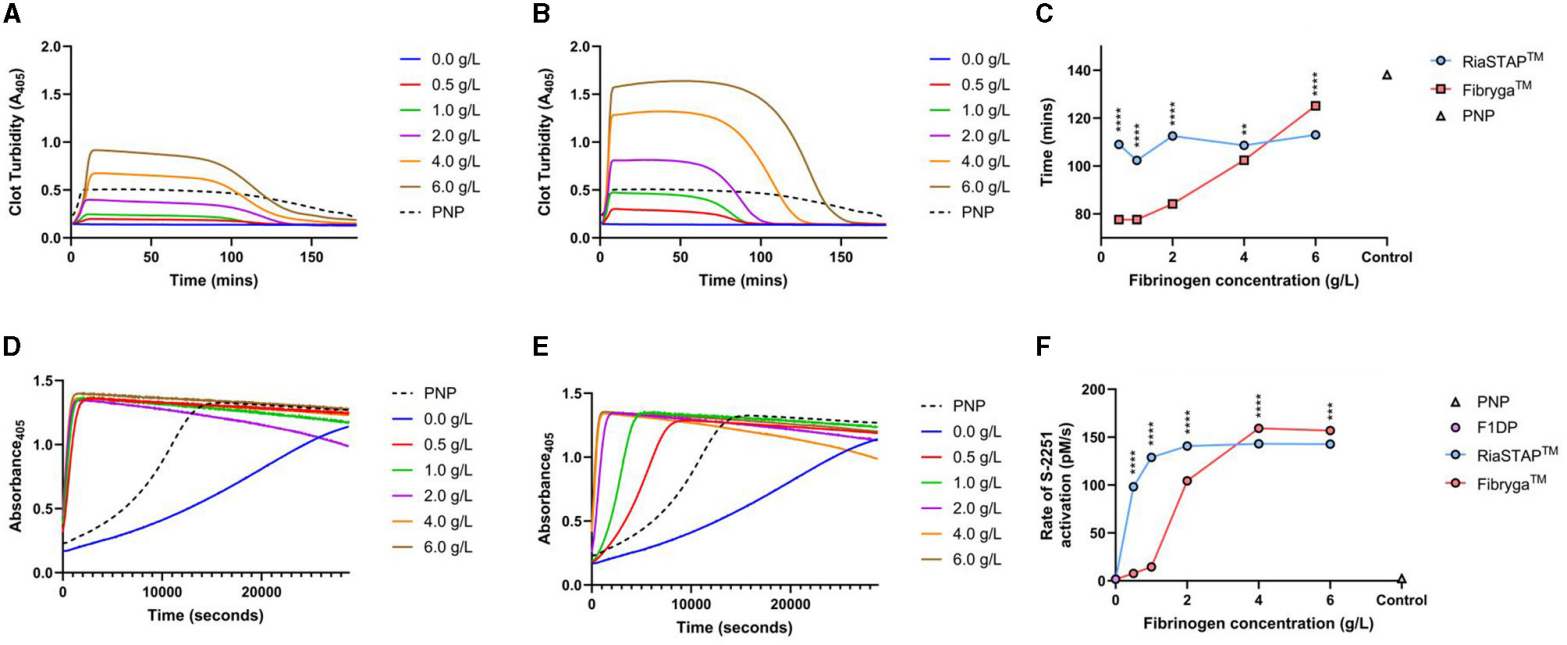
F1DP and increasing concentrations of Riastap^®^ or Fibryga^®^: clot lysis and plasmin generation, dose response curves. **(A)** RiaSTAP^®^ clot lysis curves. **(B)** Fibryga^®^ clot lysis curves. **(C)** 50% clot lysis over the five fibrinogen concentrations. **(D)** Plasmin generation curves for RiaSTAP^®^. **(E)** Plasmin generation curves for Fibryga^®^. **(F)** Plasmin generation rates over varying concentrations for RiaSTAP^®^ and Fibryga^®^. ^**^P < 0.05, ^***^P < 0.001, ^****^P < 0.0001.

Like the fibrin polymerisation experiments, reduced turbidity was seen in F1DP clot lysis assays when spiked with Riastap^®^, compared to Fibryga^®^, at the same Fg concentration. Faster lysis was seen when Fibryga^®^ was added to F1DP compared to RiaSTAP^®^ at the lower concentrations (e.g., 0.5–4 g/L) but at 6 g/L, clot lysis was faster with Fibryga^®^. Despite slower lysis, there was faster plasmin generation in RiaSTAP^®^ supplemented F1DP compared to Fibryga^®^ and notably the rate of plasmin generation plateaued at 2 g/L with Riastap^®^, but not until 4 g/L with Fibryga^®^ (Figure 4). Confocal imaging for spiked-F1DP at two concentrations (3 and 6 g/L) revealed differences (data not shown). Fibryga^®^ fibers were longer with a greater diameter at the same Fg concentration, in line with the turbidity assays.

## Discussion

We present detailed *in vivo* and *in vitro* data which explore the impact of two commercially available fibrinogen concentrates on standard laboratory coagulation tests, dynamic measures of fibrinolysis, and confocal microscopy. Our results show that in our patient with afibrinogenemia there were comparable effects of the two concentrates on measures of Fg activity and clot strength (Clauss Fg and ROTEM measures), in line with other data (13). However, there were differences seen, both in the patient samples and the *in vitro* experiments, on measures of FXIII (14) and fibrinolysis (clot lysis and plasmin generation). Confocal images confirm structural differences between the two concentrates, both in the *in vivo* samples and *in vitro* experimental F1DP samples.

In the patient, after treatment with either concentrate, clot lysis times prolonged when compared with pre-treatment, confirming both treatments increase resistance of clots to fibrinolysis. This is as expected, and in line with many other published reports (1, 11, 12, 15). However, despite similar trough Clauss Fg levels and similar recovery after the two therapies, lysis was consistently slower in the samples with Fibryga^®^ present.

Fibrin polymerization, fibrin structure and subsequent fibrinolysis are highly dependent on several variables. One important reason is the rate at which a clot is formed, which is influenced by the concentrations of thrombin and fibrinogen. High thrombin concentrations produce thinner fibrin fibers within dense networks that are less permeable and more resistant to fibrinolysis (1, 16). One of the other important factors in fibrin clot stability, and its subsequent resistance to breakdown, is the cross-linking of fibers resulting from the action of activated FXIII (17, 18).

Faster fibrin polymerization, and greater turbidity, was seen with Fibryga^®^, compared to Riastap^®^, across all Fg concentrations. These differences are important, particularly as the thrombin concentration was controlled across experiments and suggests that the structure of the fibrinogen is different between concentrates (19). Certainly, there were differences seen in the Western blot comparative analysis, with less alpha chain in the Riastap^®^. The confocal images also provide some explanation for the changes in susceptibility to lysis. After both treatments, the fibrin structure became denser and pores less numerous. The fibers after Fibryga^®^ treatment were visibly longer and the structure of the clot more readily resembled PNP. Denser fibrin structures, with fewer pores, are known to be more resistant to lysis (16, 17).

Rising concentrations of Fibryga^®^, both *in vivo* and *in vitro*, were strongly associated with longer 50% lysis times. In the patient samples, prolongation of lysis occurred in a dose dependent manner with Fibryga^®^ e.g., the Riastap^®^-Fibryga^®^ sample lysed more slowly than Riastap^®^ alone, and the longest lysis time was seen in the sample containing only Fibryga^®^ concentrate. This effect was mirrored in the F1DP-spiked plasma. Across the same concentration range of Riastap^®^, there was no important change in lysis. These data suggest that constituents within Fibryga^®^, that are absent in Riastap^®^, contribute strongly to lysis susceptibility.

Our subsequent experiments aimed to determine whether plasmin generation capacity explained the differences. Both products showed a sigmoidal dose response, with increasing plasmin generation with higher Fg concentrations. Contrary to the clot lysis experiments, the EC50 was markedly lower for Riastap^®^ compared to Fibryga^®^ (0.35 vs. 1.77 g/L). The possible reasons for more rapid plasmin generation in this experiment might be a lower A2AP or greater plasminogen concentration in Riastap^®^, although we were not able to show differences (Supplementary Table S1). Our findings require further exploration.

A notable difference between the Fg concentrates, however, is the FXIII concentration. Fibryga^®^ contains substantially more FXIII (1.24 vs. 0.01 IU/mL). Our clot lysis data both *in vivo*, and *in vitro*, show that the lysis time is affected by the type of concentrate. The Western blot data lend further strength to the notion that one of the important effectors of the difference in lytic resistance is the FXIII concentration. Our data show that RiaSTAP^®^, when exposed to thrombin, does not form γ-γ crosslinks, whereas crosslinks are evident in the Fibryga^®^ experiments; data which align with a prior publication (18). Lower quantities of FXIII crosslinks increases the susceptibility of a fibrin clot to breakdown (19, 20).

Notably, our patient has normal FXIII levels. The sample taken when he was in receipt of Riastap^®^ (e.g., no supplementary FXIII) was 0.96 IU/mL. It might be hypothesized that additional FXIII in a Fg concentrate would be of no consequence, however, our data suggest that resistance to lysis is further enhanced by Fibryga^®^. This may have important clinical consequences, particularly if a Fg concentrate were to be used for a patient with dysfibrinogenemia (where some variants confer a pro-thrombotic phenotype) or conversely, for those patients where supplementing FXIII might also be beneficial; e.g., in trauma-hemorrhage (6, 13).

Both concentrates are derived from human plasma, and some of the manufacturing steps taken may have caused structural post-translational alterations in the fibrinogen chains. The viral inactivation steps particularly are different, involving a 20-h heating process for RiaSTAP^®^ at 60°C.^3^ This compares to Fibryga^®^ which is treated with solvent-detergent for virus inactivation, and nanofiltration for virus removal (18).^4^ Denaturation of Fg chains can occur above 65°C (21).

Our data suggest that more attention should be paid to how fibrinogen concentrates are used clinically (22) and which laboratory tests are conducted to monitor therapy, e.g., should measures of fibrinolysis be considered? Notably, there are no rapid and reliable tests available to clinicians which measure mild to moderate fibrinolysis (standard ROTEM and TEG assays detect more significant lysis only) and this is an area of active laboratory research. It may be that the differences shown between these two products are not sufficiently marked to manifest as differences in clinical outcome, but our data suggest there may be room to tailor the prescription of Fg concentrate more individually. For example, for those patients in receipt of fibrinogen replacement for vascular malformations (where FXIII may also be reduced) or for those patients where fibrinolysis is an important part of the acquired coagulopathy, for example after trauma, a product with greater FXIII or greater resistance to fibrinolysis, may be preferable. Conversely, for patients with a dysfibrinogenemia genotype that confers a thrombotic risk, fibrinolytic resistance may be preferred to be avoided.

The data we present highlight that fibrinogen concentrates should not be considered entirely interchangeable, and they have differences with regards to clot stability against lysis. Further evaluation in a larger group of patients is required to answer whether these fibrinolytic changes seen in laboratory assays can be translated into important clinical outcome differences.

## Data availability statement

The raw data supporting the conclusions of this article will be made available by the authors, without undue reservation.

## Ethics statement

The studies were conducted in accordance with the local legislation and institutional requirements. The participants provided their written informed consent to participate in this study. Written informed consent was obtained from the individual(s) for the publication of any potentially identifiable images or data included in this article.

## Author contributions

SG: Data curation, Formal analysis, Investigation, Writing—original draft. JA-H: Formal analysis, Investigation, Methodology, Writing—review & editing. SH: Investigation, Validation, Writing—review & editing. DK: Conceptualization, Writing—review & editing. GM: Formal analysis, Investigation, Methodology, Supervision, Validation, Writing—review & editing. NC: Conceptualization, Methodology, Supervision, Writing—original draft, Writing—review & editing.

## Footnotes

^1^Shiny Apps for clot lysis analysis. Available online at: https://drclongstaff.shinyapps.io/clotlysisCL_2019/ (accessed January 15, 2024).

^2^Shiny Apps for plasmin generation analysis. Available online at: https://drclongstaff.shinyapps.io/zymogenactnCL/ (accessed January 15, 2024).

^3^Available at: https://riastap-prescribing-information.pdf (https://cslbehring.com) (accessed February 23, 2024).

^4^Pathogen Safety - Fibryga^®^ Fibrinogen (Human) (https://fibrygausa.com) (accessed February 23, 2024).
