# Supplementary material for: Are all fibrinogen concentrates the same? The effects of two fibrinogen therapies in an afibrinogenemic patient and in a fibrinogen deficient plasma model. A clinical and laboratory case report
Source: Front Med (Lausanne). 2024 May 30;11:1391422. doi: 10.3389/fmed.2024.1391422 (PMC11169818; doi:10.3389/fmed.2024.1391422)
Supplement: Supplementary file 1 [file Data_Sheet_1.docx]

**Supplemental Data.**

**Figure S1.**

**
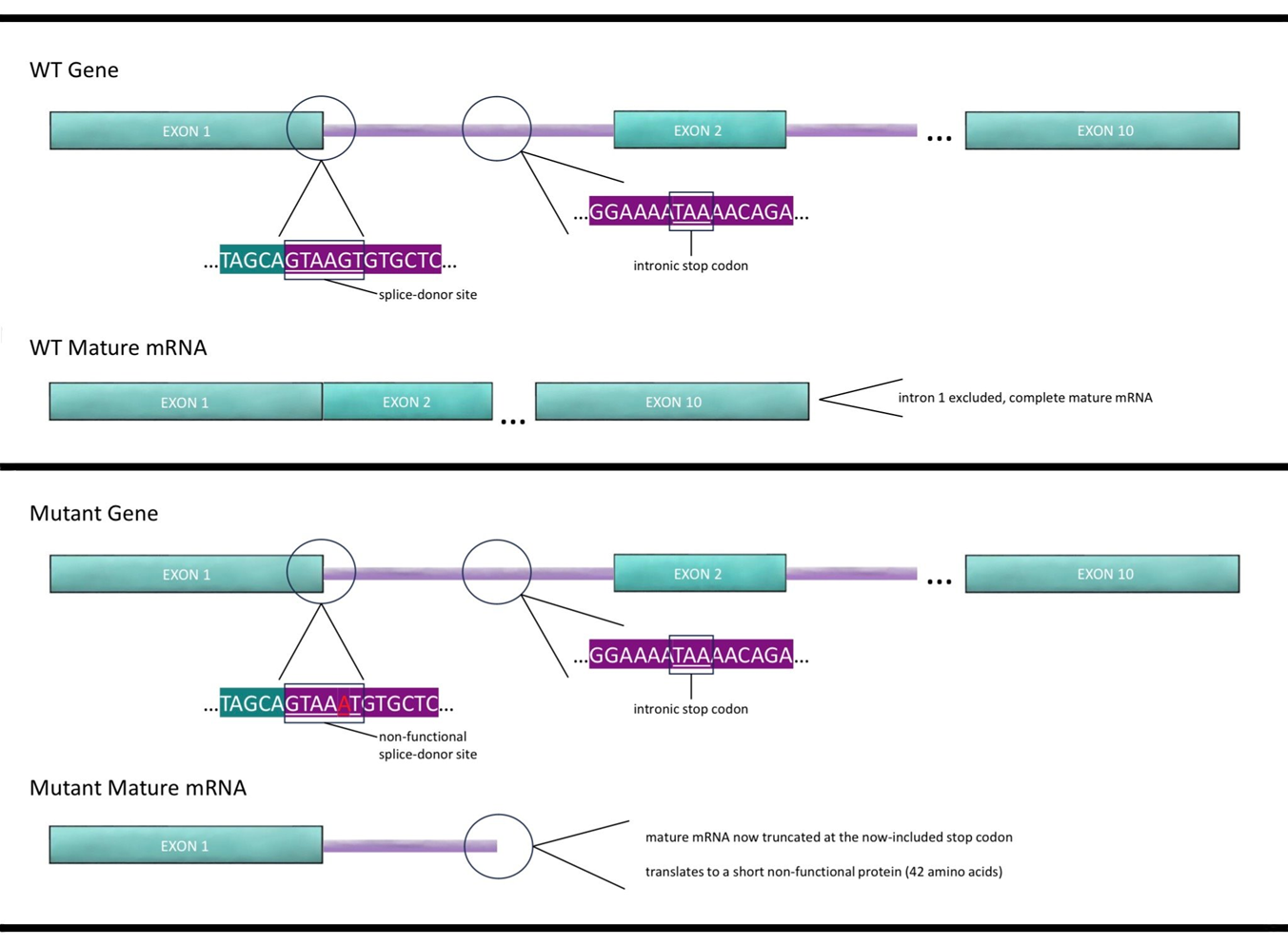
**

**Table S1.**


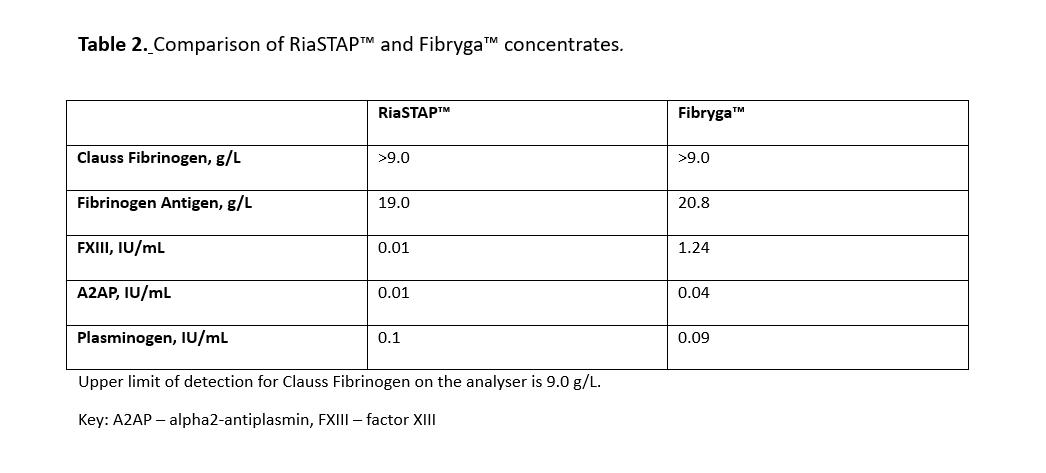


**Figure S2.**

**S2A** **S2B**


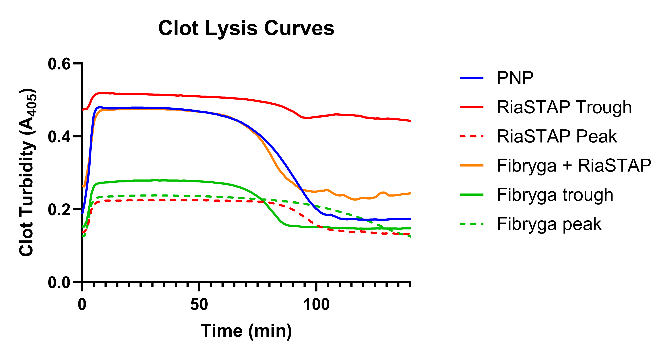

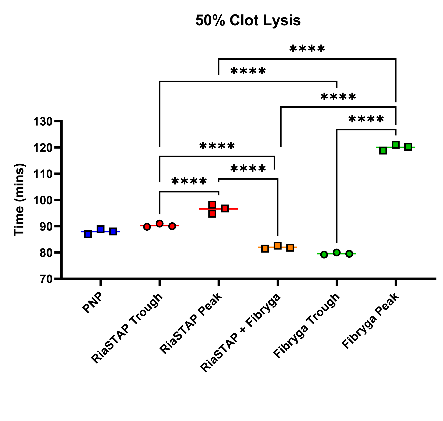


**Figure S3.**

**S3A S3B**


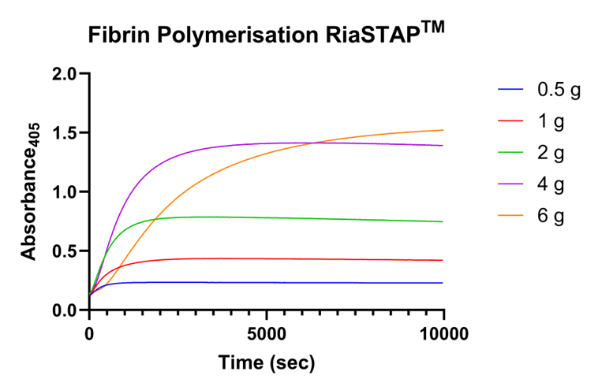

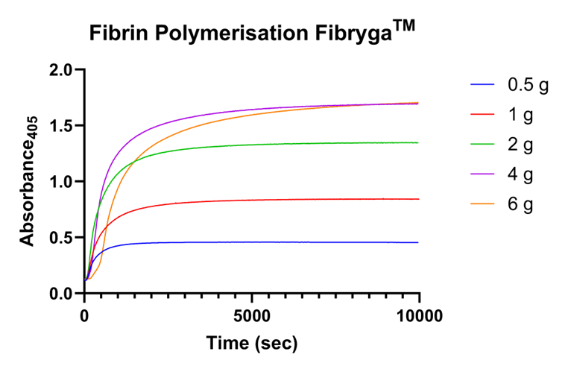


**S3C**


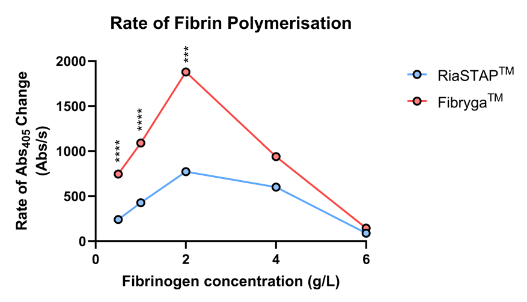


**Figure Legends for Supplemental Data**

**Figure S1.** *Fibrinogen variant.*

Our patient had a homozygous variant, c.78+5G>A: a point mutation (G>A) at the splice site of the first intron of the fibrinogen γ-chain gene, leading to the inclusion of this intron in the mature mRNA. This intron includes a stop codon, which truncates the fibrinogen protein at 42 amino acids, creating a very short and non-functional fibrinogen protein.

Key. WT – wild type

**Figure S2.** *Clot lysis curves and 50% clot lysis comparisons for patient samples.*

(S2A) Clot lysis curves. (S2B) 50% clot lysis times.

**Figure S3.** *Fibrin polymerisation, dose response curves.*

(S3A) Fibrin polymerisation, RiaSTAP®. (S3B) Fibrin polymerisation, Fibryga®. (S3C) Rate of fibrin polymerisation.
